# Whole Genome Re-sequencing Reveals Natural Variation and Adaptive Evolution of *Phytophthora sojae*

**DOI:** 10.3389/fmicb.2019.02792

**Published:** 2019-11-29

**Authors:** Xiong Zhang, Bo Liu, Fen Zou, Danyu Shen, Zhiyuan Yin, Rongbo Wang, Feng He, Yuanchao Wang, Brett M. Tyler, Wei Fan, Wanqiang Qian, Daolong Dou

**Affiliations:** ^1^Department of Plant Pathology, Nanjing Agricultural University, Nanjing, China; ^2^Department of Plant Pathology, China Agricultural University, Beijing, China; ^3^Agricultural Genomic Institute, Chinese Academy of Agricultural Sciences, Shenzhen, China; ^4^Center for Genome Research and Biocomputing, Oregon State University, Corvallis, OR, United States

**Keywords:** *Phytophthora sojae*, genome re-sequencing, natural variation, adaptive evolution, RxLR effectors, avirulent genes

## Abstract

Due to the monocultural basis of agricultural crops, mutated plant microbes with increased pathogenicity can easily spread in the field and lead to serious yield losses. As a major threat to a wide range of crop plants, oomycete pathogens continuously undergo adaptive evolution to overcome plant defense barriers. However, the genetic basis of their evolution at the molecular level remains largely unknown. Here, we investigated the nature variation and the population genomics of the soybean pathogen *Phytophthora sojae* by high-throughput genome re-sequencing. Genomic variation analysis revealed uneven “two-speed” evolutionary pattern with genes in gene-sparse regions (GSRs) showing higher rates of structural polymorphisms and positive selection. GSRs are enriched in effector genes and transposase-related genes. Our results also suggested that the NADH oxidase and MIP transporter gene families undergo rapid and diversifying selection. Furthermore, we demonstrated that *P. sojae* isolates possess varying numbers of RxLR effectors with diverse sequences, totaling 471 members. Among them, 42 core RxLR effectors are assumed to be important for infection. Finally, we observed that *Avr* genes exhibit abundant sequence variation in *P. sojae* isolates. Several novel variants lead to the evading of host resistance, including a complete deletion in *Avr3c* and amino acid mutations in *Avr1a*. Taken together, our results provide an adaptive landscape of *P. sojae* at single-nucleotide resolution, as well as resources for further resistance breeding and disease prevention against this important plant pathogen.

## Introduction

Oomycetes are fungal-like eukaryotic organisms classified into the Stramenopila kingdom, which includes multiple plant- and animal-infecting pathogens (Fawke et al., 2015). Oomycetes cause devastating diseases on a wide range of crop plants and result in severe yield loss (Jiang and Tyler, 2012). The most notorious plant-infecting oomycete pathogens belong to the genus *Phytophthora*, which includes over 100 species (Hansen et al., 2012). Being responsible for root rot in soybean (*Glycine max*), *Phytophthora sojae* is one of the most economically important *Phytophthora* pathogens (Tyler et al., 2006). As the second most destructive pathogen and a major yield-limiting factor for soybean, *P. sojae* can cause up to 100% yield loss in individual fields, resulting in around $2 billion of annual loss worldwide (Tyler, 2007). Despite the importance of *P. sojae* in soybean disease control, the molecular details of its pathogenesis and population genomics remain elusive.

Currently, the most effective way to manage disease caused by *P. sojae* is to develop cultivars harboring major resistance (*Rps*, *Resistance P. sojae*) genes (Dorrance et al., 2003). To date, over 20 *Rps* genes have been identified and mapped to nine chromosomes in soybean, including *Rps1a*, *Rps1b*, *Rps1c*, *Rps1k*, *Rps3a*, and *Rps6*. Some *Rps* genes have been incorporated into commercial soybean cultivars (Chawla et al., 2013; Huang et al., 2016). Meanwhile, 11 *Avr* genes have been identified and cloned from *P. sojae*, including *Avr1a*, *Avr1b*, *Avr1c*, *Avr1d*, *Avr1k*, *Avr3a*, *Avr3b*, Avr3c, *Avr4*, *Avr5*, and *Avr6* (Shan et al., 2004; Dong et al., 2009; Dou et al., 2010; Dong S. et al., 2011; Dong S.M. et al., 2011; Na et al., 2013; Song et al., 2013; Na et al., 2014). Among them, the *Avr3a*/*Avr5* and *Avr4/Avr6* pairs are different alleles of the same gene, respectively. All known *Avr* genes are predicted to encode effector proteins containing a secretion signal peptide followed by the RxLR (arginine-any amino acid–leucine–arginine) motif (Anderson et al., 2015). To cope with the selection pressure from soybean *Rps* genes, *P. sojae* can overcome *Rps*-mediated immune surveillance through mutations of the *Avr* genes, including amino acid changes, complete or partial deletions, frameshift, insertions and loss of transcripts (Huang et al., 2019). Continuous emergence of new *P. sojae* virulent pathotypes limits the effectiveness of *Rps* genes in the field (Schmitthenner, 1985). To prevent disease outbreaks, it is important to understand the population genomics of *P. sojae* in the field and the associated genetic variation in *Avr* genes.

Apart from their avirulent roles, *Phytophthora* RxLR effectors can play virulent roles to promote infection (Anderson et al., 2015). Every known *Phytophthora* genome harbors 100s of putative RxLR effector genes (Anderson et al., 2015). Structural studies demonstrate that the WY domain common to many RxLR type proteins possesses the core fold, which may involve in avirulence and virulence adaptation (Anderson et al., 2015). Functional studies reveal that RxLR effectors can suppress host immunity by either hijacking plant resistance pathways or utilizing plant susceptibility factors (Dou and Zhou, 2012). Comparative genomic studies indicate that most RxLR effectors are highly divergent across different pathogens (Jiang et al., 2008). Recent research has been focused on conserved effector repertoire shared by different pathogens and core effectors that are common in different strains of the same pathogen. For example, the conserved effectors between *P. sojae* and *Hyaloperonospora arabidopsidis* suppress PAMP- and effector-triggered immunity across diverse plant species (Deb et al., 2018). Four *P. infestans* core effectors contribute to virulence through defense suppression (Yin et al., 2017). Core effectors can be effective for breeding cultivars with durable resistance (Dangl et al., 2013). The re-sequencing strategy has been used to identify core effectors from 65 strains of the bacterial pathogen *Xanthomonas axonopodis* pv. *manihotis* (Bart et al., 2013). Currently we have little knowledge on core RxLR effectors of oomycete species. Identification of the core RxLR effector repertoire in *P. sojae* will be critical for screening soybean *Rps* genes conferring broad-spectrum and durable resistance.

Genome plasticity can help organisms adapt to the new environment, which is particularly the case for pathogens as they coevolve with their hosts via “arms races” (Raffaele and Kamoun, 2012; Sanchez-Vallet et al., 2018). Many filamentous microorganisms fit a typical “two-speed” genome model with distinct gene dense regions (GDRs) and gene-sparse regions (GSRs). The dynamic GSRs are enriched in rapidly evolving genes and transposons to drive adaptive evolution (Dong et al., 2015; Schrader and Schmitz, 2018). Comparative analysis of 15 *Clavicipitaceae* genomes has revealed large repeat blocks in their alkaloid biosynthesis gene loci, which can facilitate frequent gene losses, mutations, and duplications for symbiotic adaptation (Schardl et al., 2013). Comparative genomic analysis of four *Phytophthora* species has showed that their “two-speed” genome evolution is driven by host jumps. Genes in GSRs, including effector- and epigenetic-related genes, exhibit higher evolutionary rates of structural polymorphisms and positive selection (Raffaele et al., 2010). Comparative genomic analysis of four *P. sojae* isolates has revealed a relatively covert “two-speed” genome evolution, with effectors overrepresented in positive selection (Ye et al., 2016b). Despite the progress mentioned above, it remains largely unknown how the “two-speed” genome evolution model contributes to *Phytophthora* pathogenic adaptation. Considering the strong selection pressure in both nature and agricultural ecosystems (Thrall et al., 2011), population genomic analysis of *P. sojae* will enable integrated investigation of its genome plasticity and yet unknown pathogenicity determinants. In this study, we dissected the genetic basis of *P. sojae* adaptive evolution with emphasis on pathogen adaptation. We analyzed the whole genomes of 29 field *P. sojae* isolates from China and the North America. Population genomic variation analysis confirmed uneven “two-speed” evolution with genes in GSRs showing higher rates of structural polymorphisms and positive selection. In particular, both NADH oxidase and MIP transporter families are under rapid diversifying selection in GSRs. Pan-genome analysis presumed 42 putative core RxLR effectors. Moreover, *Avr* genes exhibit abundant sequence variation with several novel variants identified. Taken together, our results suggest that *P. sojae* adopts novel mechanisms for virulence, which can be a valuable resource for resistance breeding in soybean.

## Materials and Methods

### Pathogenicity Assays

All *P. sojae* isolates used in this study were routinely maintained on V8 juice agar slant at 12°C in the dark. For pathogenicity assays, all isolates were transferred to V8 juice agar plate medium at 25°C in the dark for 5 to 7 days. Their virulence was evaluated by hypocotyl inoculation (Dou et al., 2010; Song et al., 2013). Pathogenicity determination was repeated twice for each isolate. Isolates were considered as avirulent if more than 70% of the inoculated seedlings survived. Otherwise they were determined as virulent (Na et al., 2013).

### DNA Extraction, PCR Amplification, and Sequencing

Genomic DNA of *P. sojae* isolates was isolated from mycelia using the CTAB (hexadecyl trimethyl ammonium bromide) method (Murray and Thompson, 1980). Extracted DNA was quantified using the Qubit^®^ 2.0 Fluorometer kit. PCR amplifications were performed as previously described (Song et al., 2013). All primers are listed in Supplementary Table S6.

Sequencing libraries were constructed using the standard Illumina protocol. Library quality was determined using the Agilent 2100 Bioanalyzer (Agilent Technologies). All samples were sequenced using Illumina HiSeq X-ten. The paired-end reads have an average insert size of 300 bp. All read sequences were deposited to the NCBI Sequence Read Archive (SRA), under the BioProject: PRJNA578597.

### Reads Mapping and Variants Calling

Raw reads were filtered using the clean_adapter and clean_lowqual software^1^. High-quality reads account for 97.03% of the raw reads with average error rate below 0.001. They were then aligned to *P. sojae* reference genome v3.0 (Tyler et al., 2006) using the Burrows–Wheeler Transform Alignment (BWA) software package v0.7.5a (Li and Durbin, 2009). For each sample, duplicate reads were removed from alignments using samtools software package v1.8 (Li et al., 2009). The mpileup function in samtools was used to generate mpileup files for each sample. Bcftools-vcftools (Li, 2011) was used to identify SNPs and Indels in individual samples. Several criteria were applied during SNP filtering: (i) The phred quality score of base sequencing and score of read mapping higher than 60; (ii) Minimum coverage higher than 10; (iii) Allele frequency ≥ 80% for homozygous SNPs; (iv) Allele frequency between 20 and 80% for heterozygous SNPs. CNVs were estimated from read depth by the published methodology (Alkan et al., 2009). Rates of synonymous substitution (dS), non-synonymous substitution (dN) and dN/dS were calculated using KaKs_Calculator software v2.0 (Wang et al., 2010). The *P*-value for dN/dS ratio was calculated by the Fisher’s exact test. Genes with dN/dS > 1.00 and *P*-value < 0.05 were defined as under positive selection. Genes with over 1.5 kb intergenic regions were considered as GSRs (Dong et al., 2015; Chen et al., 2018). All variants were visualized by the Circos software v0.696 (Krzywinski et al., 2009).

### Population Structure Analysis

Phylogenetic analysis and principal component analysis were based on all detected SNPs. Phylogenetic tree was constructed using PhyML v3.0 (Guindon et al., 2010). Principal component analysis was conducted by SNPRelate (Zheng et al., 2012).

### RxLR Effectors Identification

Two distinct pipelines were adopted to identify RxLR effectors. The first pipeline is a reads mapping strategy with three steps: (i) Use samtools (Li et al., 2009) to extract mapped reads of each RxLR gene from re-sequenced genomes; (ii) Use SeqMan from the Lasergene software package to assemble these mapped reads; (iii) Modify the assembled sequence manually to get ORF (from start codon to stop codon) from predicted gene model. Three isolates (P7064, P7074, and P7076) were excluded from the analysis due to their low sequencing depth. All RxLR effectors identified by the first pipeline were further used to study their conservation and divergence across all isolates. Additionally, the *de novo* assembly approach (Ye et al., 2016a) was also employed to supplement the repository of RxLR effector candidates. Genomes were assembled via the SOAPdenovo assembly process (Li et al., 2010). Multiple sequence alignments were performed using MUSCLE v3.8.31 (Edgar, 2004) with default setting.

### Gene Annotation and Enrichment Analysis

Genes were annotated with PFAM terms against the Pfam database (El-Gebali et al., 2019). Hmmer software package v3.1b2 (Finn et al., 2011) was used for annotation with 1e^–5^ as the cut-off E-value. Databases for all species used in this study were obtained from their original sources: *P. capcisi* (Lamour et al., 2012), *P. infestans* (Haas et al., 2009), *P. ramorum* (Tyler et al., 2006), *H. parasitica* (Baxter et al., 2010), *P. ultimum* (Levesque et al., 2010), *S. parasitica* (Jiang et al., 2013), *A. laibachii* (Kemen et al., 2011), and *T. pseudonana* (Armbrust et al., 2004). After curation of the alignment with the G-blocks tool (Castresana, 2000), phylogenetic tree was constructed using PhyML v3.0 (Guindon et al., 2010) with 1000 bootstrap replicates.

Genes were classified as fast-evolving between any two *P. soaje* isolates with the criteria of (i) duplication or deletion event and (ii) dN/dS > 1. Gene enrichment was calculated by the formula: (Genes(g)∩Genes(c)/Genes(c))/(Genes(g)/Genes(a)), where Genes(g) is the number of genes in a family, Genes(c) is the number of fast-evolving genes or genes in GSRs, and Genes(a) is the total number of genes. With calculated value greater than 1, the family can be defined as enriched in fast-evolving genes or GSRs. The enrichment was considered to be significant with value above 2.

### Transcriptional Analysis

Transcriptome data was obtained from published literature (Wang et al., 2018). Briefly, RNA samples from mycelia and soybean leaves collected 3 h after infection were obtained as references. RNA samples from soybean leaves collected 0.5, 6, 12, 24, 36, and 48 h after infection were added. The *P. sojae* isolate P6497 and the susceptible soybean cultivar Williams were used in the stages of infection. Three biological replicates were performed for each treatment. Heatmap was generated using the Heml software (Deng et al., 2014). *Z* score normalization was calculated as (Expression_*A*_ – Mean expression_*A*__1__–__*An*_)/SD_*A*__1__–__*An*_, where Expression_*A*_ represents the expression level of a gene at one stage, Mean expression_*A*__1__–__*An*_ represents the mean expression level of a gene at all stages, and SD_*A*__1__–__*An*_ represents the standard error of the expression level of a gene at all stages.

## Results

### Virulence Assay and Genome Sequencing

Twenty-nine *P. sojae* isolates were collected from different geographical locations and investigated in this study. Using the hypocotyl split inoculation method (Song et al., 2013), the pathotypes of these isolates were determined on a set of 13 soybean lines carrying a single *Rps* gene, respectively. Twenty-eight distinct pathotypes were identified and shown in Supplementary Figure S1. Eleven and 16 isolates were able to overcome 2 to 7 and 8 to 12 *Rps* genes, respectively. The remaining two isolates were able to overcome all 13 *Rps* genes tested. These results suggest that virulence diversity is abundant in these *P. sojae* isolates.

To investigate the genetic basis of virulence diversity across *P. sojae* isolates, genome re-sequencing was performed using Illumina Hiseq X-ten PE150. A total of 93.65 Gb pair-end (PE) data were generated from 25 *P. sojae* isolates with sequencing depth of 27.9 to 51.4X coverage (Supplementary Table S1). Three published *P. sojae* genomes were obtained from eumicrobedb.org (formerly VBI Microbial Database) with sequencing depth ranging from 6.8 to 13.2X coverage (Wang et al., 2011). After removing low-quality reads, approximate 83.98 Gb high-quality PE reads were mapped to the reference genome V3.0 of *P. sojae* (Tyler et al., 2006). Including the reference, a total of 29 isolates were used for further studies below.

### Genomic Variation and Population Structure in *P. sojae*

**FIGURE 1:**
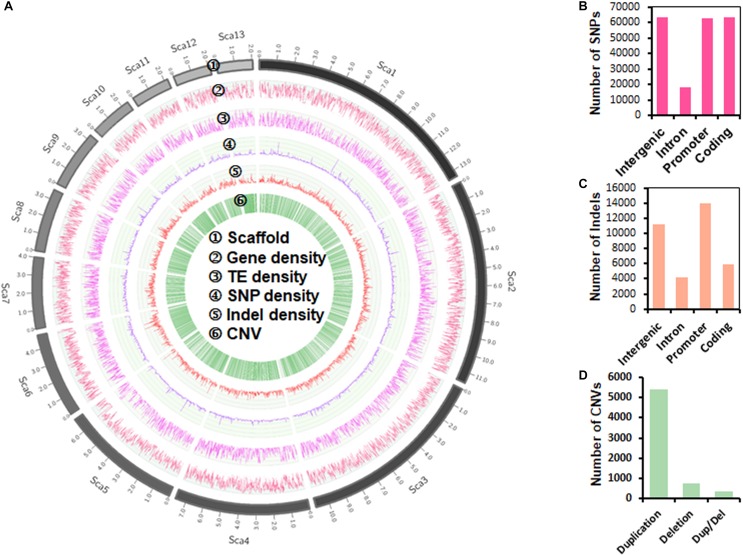
Genome-wide landscape of genetic variation in *P. sojae.* **(A)** Circos plot of the top 13 longest scaffolds (90.25% coverage of the *P. sojae* genome). Tracks from outside to inside are: ➀ scaffolds (Sca) number; ➁–➄ density of gene, transposable element, SNP (single-nucleotide polymorphism) and Indel (insertion or deletion) in non-overlapping 10 kb windows; ➅ CNV (copy number variation) location. **(B)** Distribution of SNPs in different gene regions. **(C)** Distribution of Indels in different gene regions. **(D)** Categories of CNVs.

Genomic sequence variations, including single-nucleotide polymorphism (SNP), insertion or deletion (Indel), and copy-number variation (CNV), were cataloged using a read-mapping strategy against the *P. sojae* reference genome (Tyler et al., 2006). We detected 207,740 SNPs, 35,324 Indels, and 6,573 CNVs (Figure 1A). The proportions of SNPs identified in intergenic, intron, promoter, and coding regions were 30.45, 8.76, 30.16, and 30.63%, respectively (Figure 1B). 24,311 synonymous, 39,317 missenses, and 1,168 nonsense SNPs were detected from the coding sequences. The 35,324 Indels identified ranged from 1 to 106 bp in length (Supplementary Figure S2), with the majority (86.15%) shorter than 12 bp and low proportion (4.60%) of long (>20 bp) sequences. The proportions of Indels in intergenic, intron, promoter, and coding regions were 31.78, 11.86, 39.62, and 16.74%, respectively (Figure 1C). Most (4,445) coding-region Indels caused frame-shift mutations, reflecting the influence of positive selection. The 6,573 CNVs characterized in coding genes included 5,423 duplications, 776 deletions and 374 duplications/deletions (Figure 1D).

**FIGURE 2:**
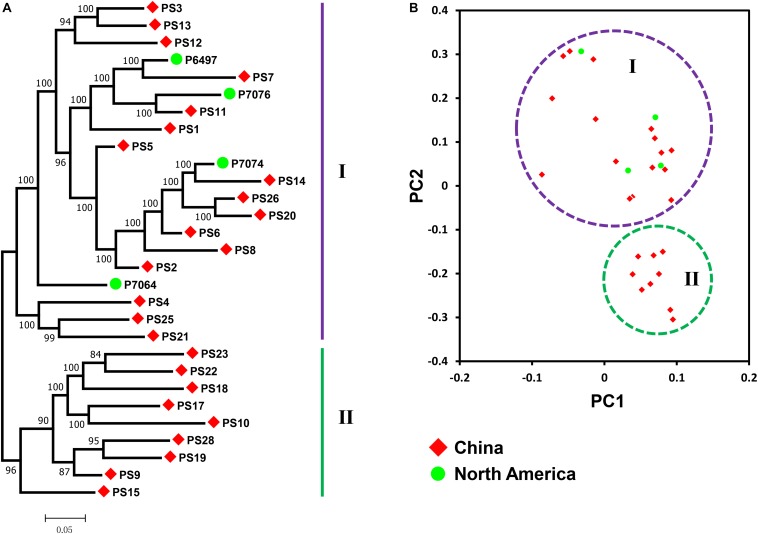
Population structure in *P. sojae*. **(A)** Phylogenetic analysis of *P. sojae* populations with all detected SNPs. The maximum likelihood tree was constructed using PhyML v3.0 with 1000 bootstrap replicates. Groups I and II are designated using the purple and green lines, respectively. **(B)** Principal component (PC) analysis of *P. sojae* populations with all detected SNPs. Principal component analysis was performed by SNPRelate. Groups I and II are encircled using the purple and green circles, respectively. Red diamonds represent isolates collected from China. Green circles represent isolates collected from North America.

To investigate the phylogenetic relationship of *P. sojae* populations, a phylogenetic tree was first constructed with detected 207,740 SNPs among the 29 isolates. Results showed no clustering of isolates by geography. We found that isolates from China could be roughly divided into two groups (Figure 2A). Group I was clustered with all four isolates from North America, and group II contained isolates only form China, indicating that isolates from China might not be part of the same clonal lineage. Principal component analysis also got the similar results (Figure 2B).

### Genomic Variation Uncovers Adaptive Evolution in *P. sojae* Populations

To explore whether distinct genomic regions evolve at significantly different rates, we calculated and compared genomic variations in GSRs and GDRs, including SNP and Indel densities per kb, CNVs and dN/dS ratios in genes (ORFs) (Supplementary Figure S3A). Compared to GDRs, GSRs showed significantly higher densities of both SNP and Indel. Averages of CNVs and dN/dS ratios were also significantly higher in GSRs than in GDRs. Moreover, we compared the transcription induction levels at seven stages versus the mycelia stage (Wang et al., 2018). Genes in GSRs showed significantly higher induction levels than those in GDRs within 24 h of soybean leave inoculation (Supplementary Figure S3B), implicating the adaptation of *P. sojae* genome evolution to plant infection.

**FIGURE 3:**
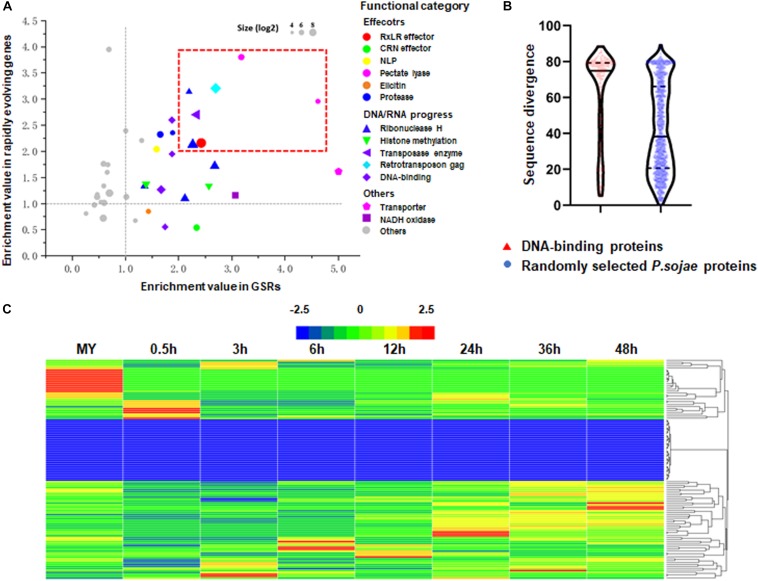
High correlation of genes density and evolving speed. **(A)** Enrichment index in GSRs (*x* axis) and rapidly evolving genes (*y* axis). The top 40 enrichment gene families are shown as the indicated colored bubbles. Bubble sizes are proportional to the sizes of gene families. **(B)** Violin plot visualization of sequence divergence of genes encoding DNA-binding proteins and randomly selected genes between *P. sojae* and *P. ramorum*. Gene sequence divergence is defined as one minus sequence identity. Red bar indicates genes encoding DNA-binding proteins. Blue bar indicates randomly selected genes as controls. **(C)** Hierarchical clustering of transcriptional patterns for genes encoding DNA-binding proteins in *P. sojae. Z* score normalization was applied for the transcription levels of genes at all stages. Cluster analysis was performed using the Heml software.

Using previously reported method (Raffaele et al., 2010), we performed domain enrichment analysis on predicted proteomes encoded by rapidly evolving genes and genes in GSRs, respectively. A total of 274 domains met the criteria of appearing in 10 or more proteins. We demonstrated that 27 and 33 domains were statistically enriched in proteomes encoded by rapidly evolving genes and GSRs, respectively. There were 23 domains shared by both groups (Figure 3A). As expected, some RxLR effectors, pectate lyase, NLP and protease were enriched in both groups whereas CRN effectors and elicitins were only enriched in the proteome encoded by rapidly evolving genes. Some domains, such as transposase, retrotransposon gag, and ribonuclease H, were also enriched in both groups. Interestingly, we found that histone methylation and DNA-binding domains, which were thought to be key regulators of gene expression (Brennan and Matthews, 1989; Raffaele et al., 2010), were enriched in the proteome encoded by rapidly evolving genes. We then calculated the pairwise sequence similarity of DNA-binding proteins between two related species *P. sojae* and *P. ramorum*. The highest sequence identity within any given DNA-binding protein paralogs was only 38.18%, whilst the average sequence identity of 1,000 randomly selected protein paralogs was 69.27%. The result indicates that DNA-binding proteins have above-average divergence levels (Figure 3B). Transcriptome analysis further revealed that most of their encoding genes exhibited stage-specific expression patterns (Figure 3C). Among them, 20 and 141 genes exhibited developmental- and infection-specific expression patterns, respectively.

**FIGURE 4:**
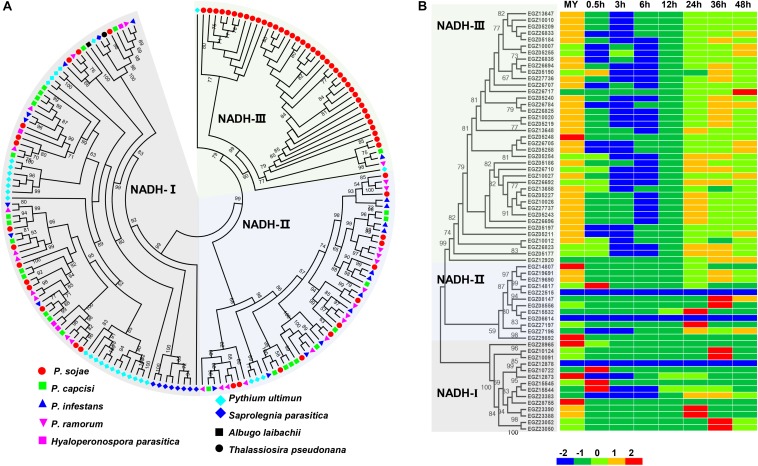
Member expansion of the NADH oxidase gene family in *P. sojae*. **(A)** Phylogenetic analysis of oomycete NADH oxidase genes. Nine indicated species were used for analysis. The maximum likelihood tree was constructed using PhyML v3.0 with 1000 bootstrap replicates. Bootstrap values higher than 50% are shown. The accession numbers of sequences used in the analysis are available in Supplementary Table S2. **(B)** Phylogenetic analysis and transcriptional patterns of NADH oxidase genes in *P. sojae*. *Z* score normalization was applied for transcription levels at all stages.

NADH oxidases play key roles in the development and virulence of filamentous fungi (Segmuller et al., 2008; Heller and Tudzynski, 2011; Liu et al., 2014). MIP transporters are potential targets of antifungal drugs against pathogenic fungi (Verma et al., 2014). Surprisingly, domains annotated as NADH oxidase and MIP transporter families were significantly enriched in the proteome encoded by rapidly evolving genes. Further analysis revealed that the NADH oxidase gene family expanded in *P. sojae* when compared to other oomycetes (Figure 4A and Supplementary Table S2). We identified 65 NADH oxidase genes in *P. sojae* and 26, 20, 27, 4, 29, 9, 1, and 1 homologous genes in *P. capcisi*, *P. infestans*, *P. ramorum*, *H. parasitica*, *Pythium ultimum*, *Saprolegnia parasitica*, *Albugo laibachii*, and *Thalassiosira pseudonana*, respectively. Gene expansion in *P. sojae* was mainly found in the NADH-III clade. Transcriptome analysis revealed that 38 NADH oxidase genes showed highest expression at the mycelia stage and 58 genes of this family were downregulated during the early biotrophic phase (Figure 4B). Additionally, we identified 35, 25, 22, 35, 4, 11, 5, 2, and 2 MIP genes in *P. sojae*, *P. capcisi*, *P. infestans*, *P. ramorum*, *H. parasitica*, *P. ultimum*, *S. parasitica*, *A. laibachii*, and *T. pseudonana*, respectively (Supplementary Table S3). Unlike NADH oxidase genes, MIPs had no significant expansion in *P. sojae*.

### The *P. sojae* RxLR Effector Repertoire

**FIGURE 5:**
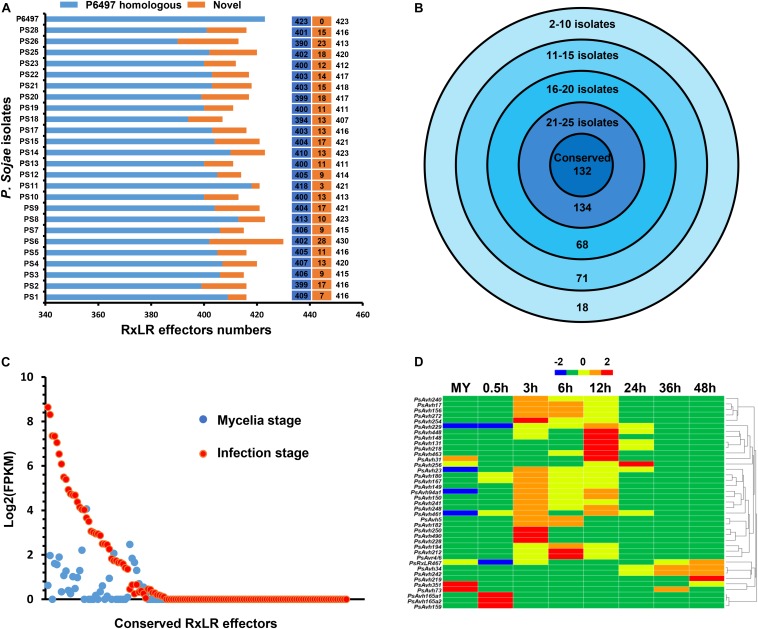
The RxLR effector repertoire in *P. sojae*. **(A)** Number of RxLR effectors predicted in 26 *P. sojae* isolates. Blue color indicates homologs of known RxLR effectors. Yellow color indicates predicted novel RxLR effectors. **(B)** Distribution of RxLR effectors across 26 *P. sojae* isolates. Number of species in which certain effectors were exclusively found is indicated at the outermost circle, and number of effectors in the set is indicated at the bottom. The innermost circle represents conserved effectors found in all 26 *P. sojae* isolates. **(C)** The FPKM values in mycelia and highest FPKM values at infection stages of 132 conserved RxLR effectors. **(D)** Hierarchical clustering of the transcriptional patterns of 42 core RxLR effectors in *P. sojae*. *Z* score normalization was applied for the transcription levels at various stages.

During virulence and colonization, *Phytophthora* pathogens target different subcellular compartments of the plant cell via deploying a large number of RxLR effectors (Jiang and Tyler, 2012; Anderson et al., 2015; Qiao et al., 2015; Kong et al., 2017). Genome sequencing unveiled 374 RxLR effectors in *P. sojae* isolate P6497 previously (Jiang et al., 2008). In the updated of reference genome of *P. sojae* isolate P6497, a total of 423 RxLR effectors were obtained in our re-identification. Considering the low sequencing depth of *P. sojae* isolates P7064, P7074 and P7076, we used the remaining 25 isolates for RxLR effector analysis. First, we identified 390 to 418 homogeneous RxLR effectors in 25 isolates using the reads mapping approach (Figure 5A). In addition, we identified 3 to 28 novel RxLR effectors in 25 isolates using the *de novo* assembly approach (Figure 5A). Altogether, the numbers of RxLR effectors ranged from 407 to 430 in these isolates (Figure 5A). There were totally 471 RxLR effectors across isolates with 97 novel ones being identified for the first time.

Next, we investigated the conservation and divergence of the RxLR effectors that identified by reads mapping across *P. sojae* populations. Generally, the RxLR effectors were highly divergent in *P. sojae* isolates. There were 157 RxLR effectors that could be found in no more than 20 *P. sojae* isolates (Figure 5B). In contrast, 132 conserved RxLR effectors existed in all 26 isolates with 100% similarity (Figure 5B). In particular, 42 conserved RxLR effectors had detectable expression (FPKM > = 2) at one or more stages of infection (Figures 5C,D and Supplementary Table S4), indicating their roles in virulence. We considered these 42 RxLR effectors as core effectors based on their sequence and transcriptional patterns. Among them, PsAvh241 and PsAvh23 have been demonstrated to be essential for the full virulence of *P. sojae* (Yu et al., 2012; Kong et al., 2017). The other 40 core effectors may also play critical roles in the infection process.

### *Avr* Genes Exhibit Abundant Sequence Variation

**FIGURE 6:**
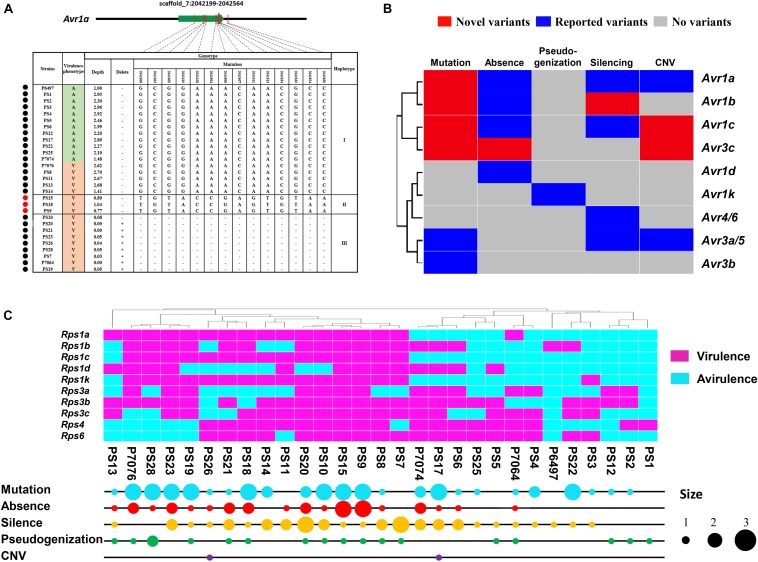
*Avr* genes exhibit abundant sequence variation among 29 *P. sojae* isolates. **(A)** Structural and nucleotide diversity at the *Avr1a* locus among 29 *P. sojae* isolates. The green arrow indicates gene-coding region. Red vertical lines indicate variant regions of the gene. Variant positions and types are connected by dotted lines. Schematic graphs of the variant positions in all isolates are grouped by haplotypes. Black circles indicate previously reported haplotypes and corresponding phenotypes. Red circles indicate novel haplotypes and corresponding phenotypes. Disease outcomes on soybean plants harboring *Rps1a* are recorded as avirulent (A) or virulent (V). Variation positions are from *P. sojae* reference genome V3.0. **(B)** Summary of different variant types of the 9 *Avr* genes. *X* axis indicates variant types of corresponding *Avr* genes. “Novel variants” indicates new variant types identified in this study. “Reported variants” indicates variant types previously described. “No variants” indicates no variant being found. *Y* axis denotes cluster analysis generated by the Heml software using the variant types of *Avr* genes. **(C)** Summary of *Avr* variant types and virulence formulas across 29 *P. sojae* isolates. Bubble sizes are proportional to the numbers of corresponding variant types. Cluster analysis was generated by Heml using the virulence formulas of *P. sojae*.

The gene-for-gene resistance to *Phytophthora* is often overcome by the rapid evolution of *Avr* genes under field conditions. To date, nine *Avr* genes recognized by 11 cognate soybean *R* genes have been cloned in *P. sojae* (Anderson et al., 2015). To better understand the gene-for-gene interactions between *P. sojae* and soybean, we examined 261 (29^∗^9) interaction combinations between 29 *P. sojae* isolates and 9 *Rps* genes. Among them, 182 interactions confirmed previous studies on *Avr* haplotypes and associated phenotypes. Taking *Avr1a* as an example, 12 isolates harbored the *Avr* haplotype and the other 17 isolates exhibited three different virulent haplotypes. Five virulent isolates contained *Avr1a-I* haplotype, which may undergo gene silencing to escape the recognition of *Rps1a*. Nine virulent isolates lost *Avr1a*. Both haplotypes were consistent to previous report (Na et al., 2014). Interestingly, we found the three isolates left constituted a novel *Avr1a* haplotype (named as *Avr1a-II*). In this haplotype, substitution of 14 nucleotides led to the mutation of 9 amino acid residues (Figure 6A). Taken together, 17 new interactions were identified in 4 *Avr* genes and their corresponding haplotypes (Figure 6B and Supplementary Table S5). Among them, 15 newly discovered homozygous *Avr* haplotypes were verified by PCR amplification and sequencing (Supplementary Table S5).

We summarized five strategies for overcoming gene-for-gene resistance in *P. sojae*, including amino acid mutation, gene absence, pseudogenization, gene silencing, and CNV (Figure 6B). Among them, amino acid mutation was the most common escape mechanism. *Avr1b*, *Avr1c*, and *Avr3c* all harbored new virulent alleles (Figure 6B and Supplementary Figures S4–S6). In contrast, pseudogenization of *Avr* genes is rare and only in response to *Avr1k* (Song et al., 2013). Seven haplotypes were detected in *Avr1b*. *Avr1b-II* showed a nucleotide substitution at -2^*nd*^ base upstream of the start codon in three virulent isolates. Avr1b-VI exhibited two-amino-acid polymorphisms in two virulent isolates (Supplementary Figure S4). There were also seven haplotypes detected in *Avr1c* (Supplementary Figure S5). In four virulent isolates, Avr1c-IV showed five-amino-acid polymorphisms resulted from substitution of 20 nucleotides. Interestingly, *Avr1c-III* was heterozygous with one avirulent copy of *Avr1c-I*. For *Avr3c*, six haplotypes were identified (Supplementary Figure S6). *Avr3c-VI* showed a complete deletion in one virulent isolate. *Avr3c-V* displayed 14^*th*^-amino-acid polymorphisms caused by substitution of 19 nucleotides in one virulent isolate. Avr3c-I showed 10-amino-acid polymorphisms mediated by substitution of 12 nucleotides in one avirulent isolate.

*Phytophthora sojae* isolates exhibited distinct evolution themes. For example, isolates PS9 and PS15 could overcome all 11 *Rps* genes. Four evolutionary mechanisms, including amino acid mutation, gene absence, pseudogenization and gene silencing, existed in these two isolates with the first two being dominant (Figure 6C). Isolates PS1 and P6497, which overcame only one *Rps*, showed either pseudogenization or gene silencing (Figure 6C).

## Discussion

Plant diseases caused by *Phytophthora* are a major threat to crop production. However, little is known about the genetic basis of adaptive evolution, especially pathogen adaptation, in *Phytophthora*. In this study, we sequence the genomes of 28 field *P. sojae* isolates and compare them to the reference genome.

Previous studies indicate that several *Phytophthora* genomes contain a typical “two-speed” architecture, with GSRs being more plastic than GDRs and serving as a cradle for adaptive evolution (Raffaele and Kamoun, 2012; Dong et al., 2015). We confirm that genomic variation in *P. sojae* shows uneven “two-speed” evolutionary rates with GSRs experiencing accelerated evolution. This observation is consistent with previously reported comparative genome analysis results of four *Phytophthora* clade 1c species (Raffaele et al., 2010). The “two-speed” strategy for driving adaptive evolution may be conserved across *Phytophthora* species. Under strong selection pressures from plant immune systems and the environment (Croll and McDonald, 2017), many pathogen genes tend to evolve rapidly for various adaptation. Consistent with previous analysis on four *Phytophthora* clade 1c species (Raffaele et al., 2010), *P. sojae* GSRs are enriched in transposons and rapidly evolving genes encoding virulence effectors, cell wall hydrolases, transposon-related proteins, and proteins related to epigenetic maintenance, suggesting the existence of a conserved *Phytophthora* adaptation and pathogenicity strategy. Interestingly, *P. sojae* GSRs are also enriched in genes encoding NADH oxidases and MIP transporters. NADH oxidases are involved in sexual differentiation (Malagnac et al., 2004), virulence (Segmuller et al., 2008), cell degeneration and host defense (Haedens et al., 2005). MIP transporters are involved in a wide range of cellular processes in human (Preston et al., 1992), plant (Wong et al., 2018) and protozoan parasites (Dean et al., 2014). They are attractive targets for antifungal drugs in pathogenic fungi (Verma et al., 2014). No NADH oxidase or MIP transporter has been functionally investigated in *Phytophthora*. They can be promising targets for future research.

Effectors, especially highly-conserved core effectors, have become powerful tools to develop disease resistance cultivars against various pathogens (Dangl et al., 2013; Vleeshouwers and Oliver, 2014). For example, by referring to an effector repertoire predicted from the *P. infestans* genome, two cognate *Rps* genes (*Rpi-sto1* and *Rpi-pta1*) were rapidly cloned from incompatible *Solanum* species (Vleeshouwers et al., 2008). Unfortunately, little is known about the core effectors in *P. sojae* to date. In this study, we identify a set of 471 RxLR effectors across 26 *P. sojae* genomes. We demonstrate that *P. sojae* harbors a significantly larger RxLR repertoire than other species. Being conserved in protein coding sequences and expressed at one or more infection stages, 42 members are defined as core RxLR effectors. Core effectors are generally considered important to pathogen virulence (Dangl et al., 2013). For example, PsAvh241 and PsAvh23 are two core effectors that are essential for full virulence of *P. sojae* (Yu et al., 2012; Kong et al., 2017). Transcriptome comparison uncovers 18 core RxLR effectors from five *P*. *infestans* isolates. Nine of them contribute to virulence through defense suppression (Yin et al., 2017). Soybean Rps proteins may recognize certain core effectors to activate defense against *P. sojae* isolates. Our RxLR effector repertoire provides a valuable resource for the search of novel *Rps* genes.

Nine *Avr* genes have been cloned and their gain-of-virulence alleles have been analyzed in *P. sojae* (Anderson et al., 2015). However, little is known about their polymorphism across *P. sojae* populations and interactions with *Rps* genes. In this study, we examine *Avr* polymorphism as well as haplotype-phenotype matches in 261 interactions between 9 *Rps* genes and 29 *P. sojae* isolates. Recently, re-sequencing of 31 *P. sojae* isolates has revealed stable predictive markers and variants for five *Avr* genes (Arsenault-Labrecque et al., 2018). Our results not only confirm these existing markers but also identify new markers for a broader range of *Avr* genes. Inexplicably, unexpected phenotypic outcomes were often observed when taking into account *Avr* haplotypes and corresponding phenotype. Previous study has revealed that gene silencing and epistatic effects might confuse the phenotypic interactions (Qutob et al., 2013; Arsenault-Labrecque et al., 2018). Nevertheless, these inexplicable interactions require further investigation.

In our study, Avr effectors containing new amino acid polymorphisms will offer new resources for functional characterizations of *Avr* genes in plant–pathogen interactions. Meanwhile, CNV and gene silencing raise new challenges to traditional PCR detection of *Avr* genes. Other methods, such as RT-PCR, are needed to measure the transcript levels and CNVs of *Avr* genes. 26 out of 29 collected isolates can overcome *Rps5*. In contrast, only 13 isolates can overcome *Rps1d*, indicating that *Rps1d* is overall more effective for controlling *Phytophthora* diseases. This result is consistent with a previous report by Moriwaki (2010). No *Rps* gene can render resistance to all 29 isolates in our test. Therefore, efforts should be made to identify novel *Rps* genes conferring durable and broad-spectrum resistance. The core RxLR effector repertoire we report can play a key role in the *Rps* search.

In recent years, genome re-sequencing has emerged as a powerful tool for dissecting the genetic basis of trait adaptation in fungal pathogens, such as virulence, lifestyles, fungicide resistance, host jump, and host specialization (Grunwald et al., 2016). However, few cases have been reported in oomycete pathogens. In this study, we re-sequence the genomes of 26 field *P. sojae* isolates. Our comparative genomic analysis results provide new clues for adaptive evolution, especially pathogen adaptation, in *Phytophthora*. We demonstrate that *P. sojae* genome undergoes uneven “two-speed” evolution with genes in GSRs exhibiting higher rates of sequence polymorphism and positive selection. GSRs are enriched by effector genes, transposase-related genes and gene families encoding NADH oxidases and MIP transporters. In addition, we report an updated RxLR effector repertoire with 42 core effectors. Finally, we show that *Avr* genes exhibit abundant sequence variation and present several novel variants. Our work provides a genomic platform for studying soybean-*Phytophthora* interaction and breeding new resistant cultivars.

## Data Availability Statement

All read sequences for this study were deposited to the NCBI Sequence Read Archive (SRA), under the BioProject: PRJNA578597 (accession numbers: SAMN13066420, SAMN13066421, SAMN13066422, SAMN13066423, SAMN13066424, SAMN13066425, SAMN13066426, SAMN13066427, SAMN13066428, SAMN13066429, SAMN13066430, SAMN13066431, SAMN13066432, SAMN13066433, SAMN13066434, SAMN13066435, SAMN13066436, SAMN13066437, SAMN13066438, SAMN13066439, SAMN13066440, SAMN13066441, SAMN13066442, SAMN13066443, and SAMN13066444).

## Author Contributions

DD, WF, WQ, and BT directed the project. XZ, BL, DS, ZY, and WF analyzed the data. FZ, RW, and FH performed the experiments. YW, BT, and WQ provided very critical suggestions. DD and XZ wrote the manuscript with input from all other authors.

## Conflict of Interest

The authors declare that the research was conducted in the absence of any commercial or financial relationships that could be construed as a potential conflict of interest. The handling Editor declared a past collaboration with one of the authors BT.

## Footnotes

1 https://github.com/fanagislab/common_use
