# Supplementary figures and images for: Whole Genome Re-sequencing Reveals Natural Variation and Adaptive Evolution of Phytophthora sojae
Source: Front Microbiol. 2019 Nov 29;10:2792. doi: 10.3389/fmicb.2019.02792 (PMC6895562; doi:10.3389/fmicb.2019.02792)

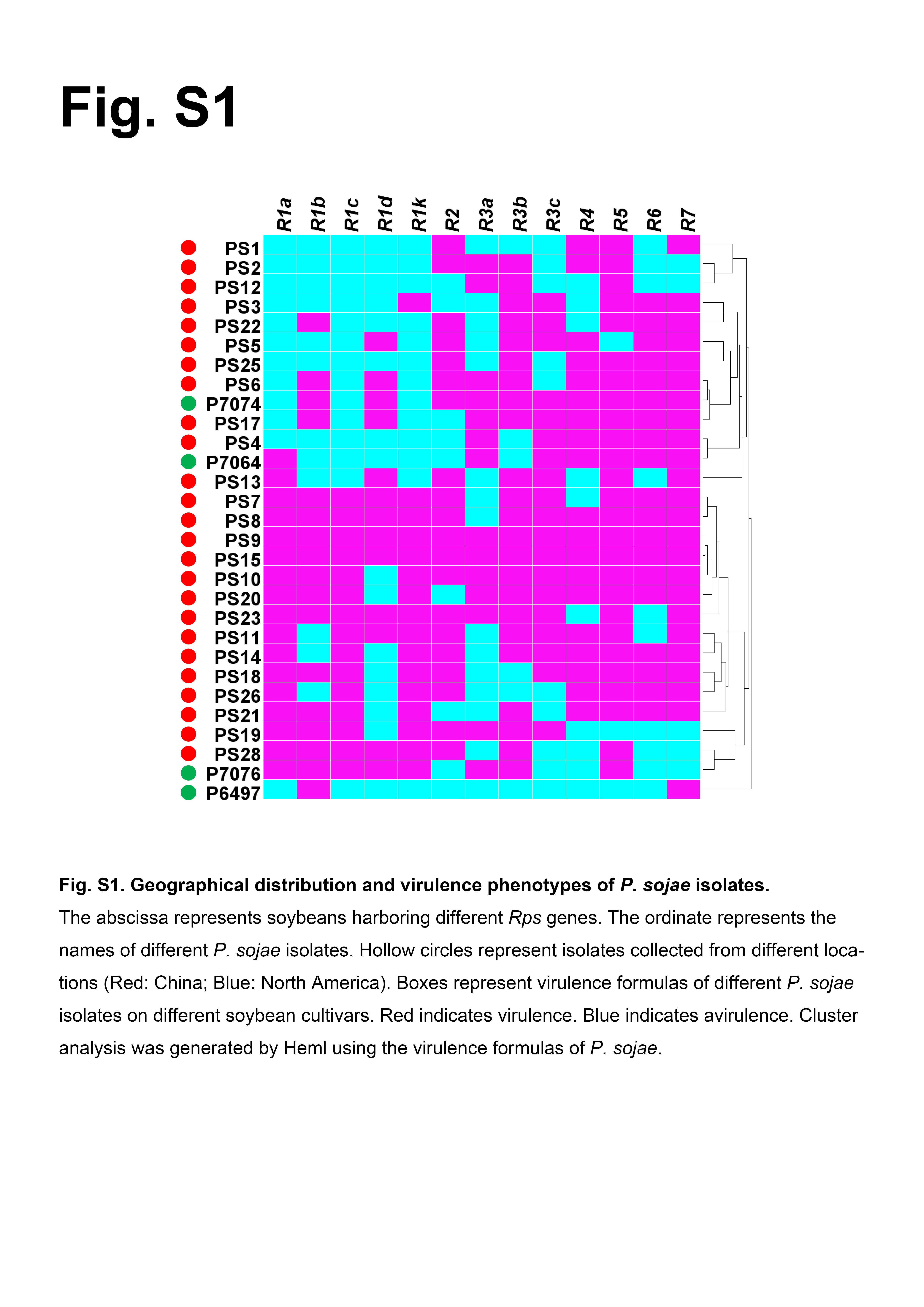

Supplement: Supplementary file 1 [file Image_1.TIF]

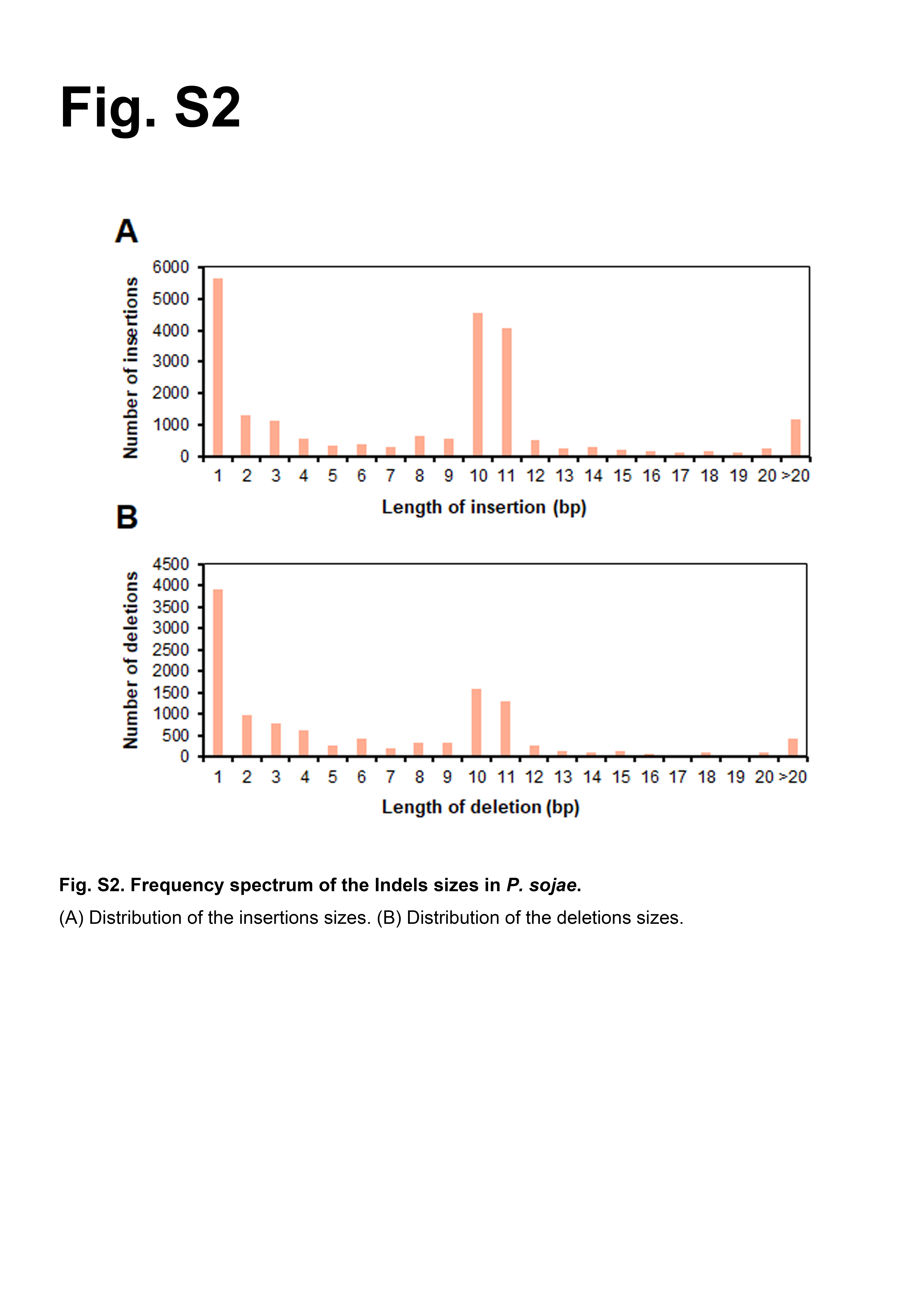

Supplement: Supplementary file 2 [file Image_2.TIF]

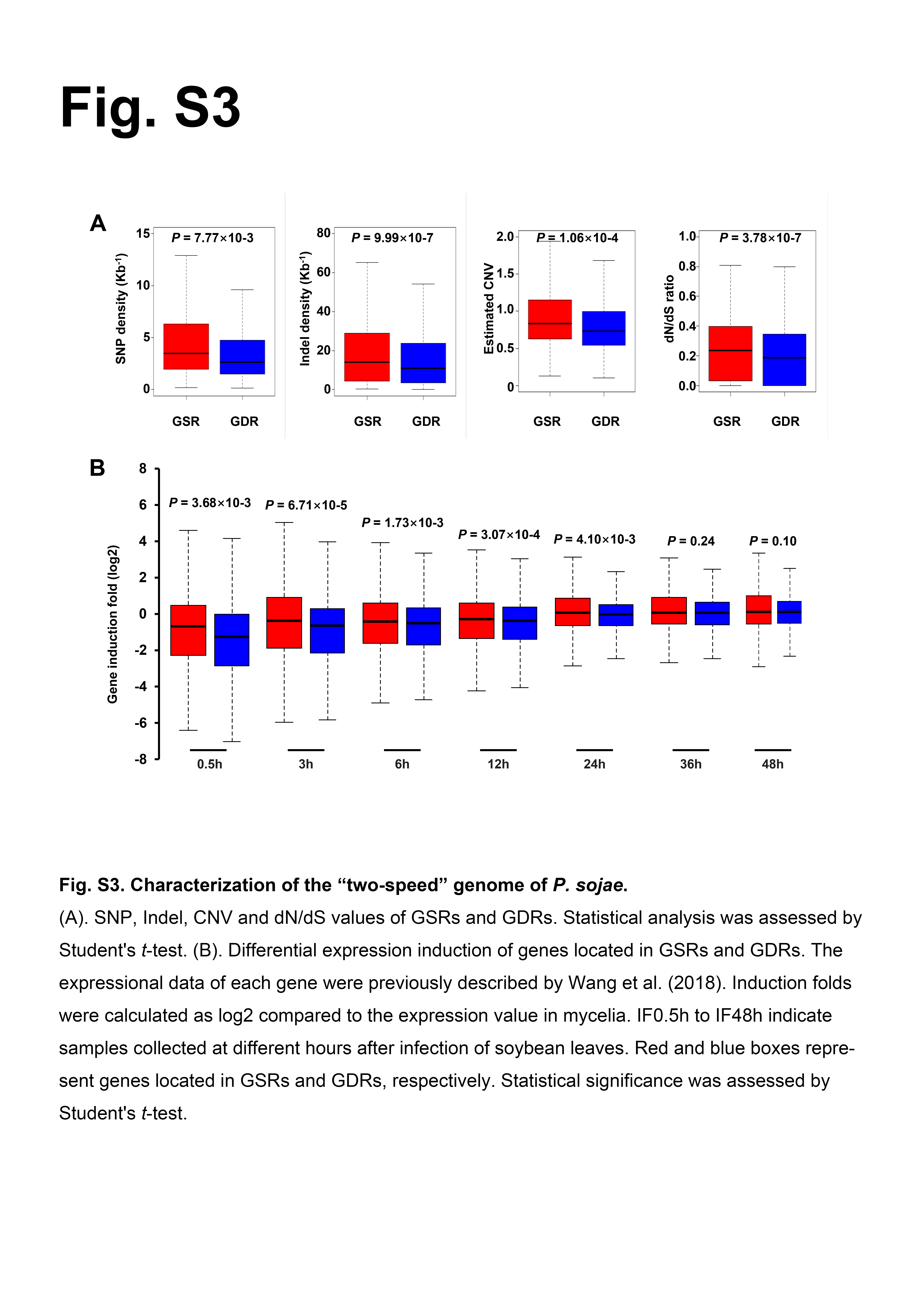

Supplement: Supplementary file 3 [file Image_3.TIF]

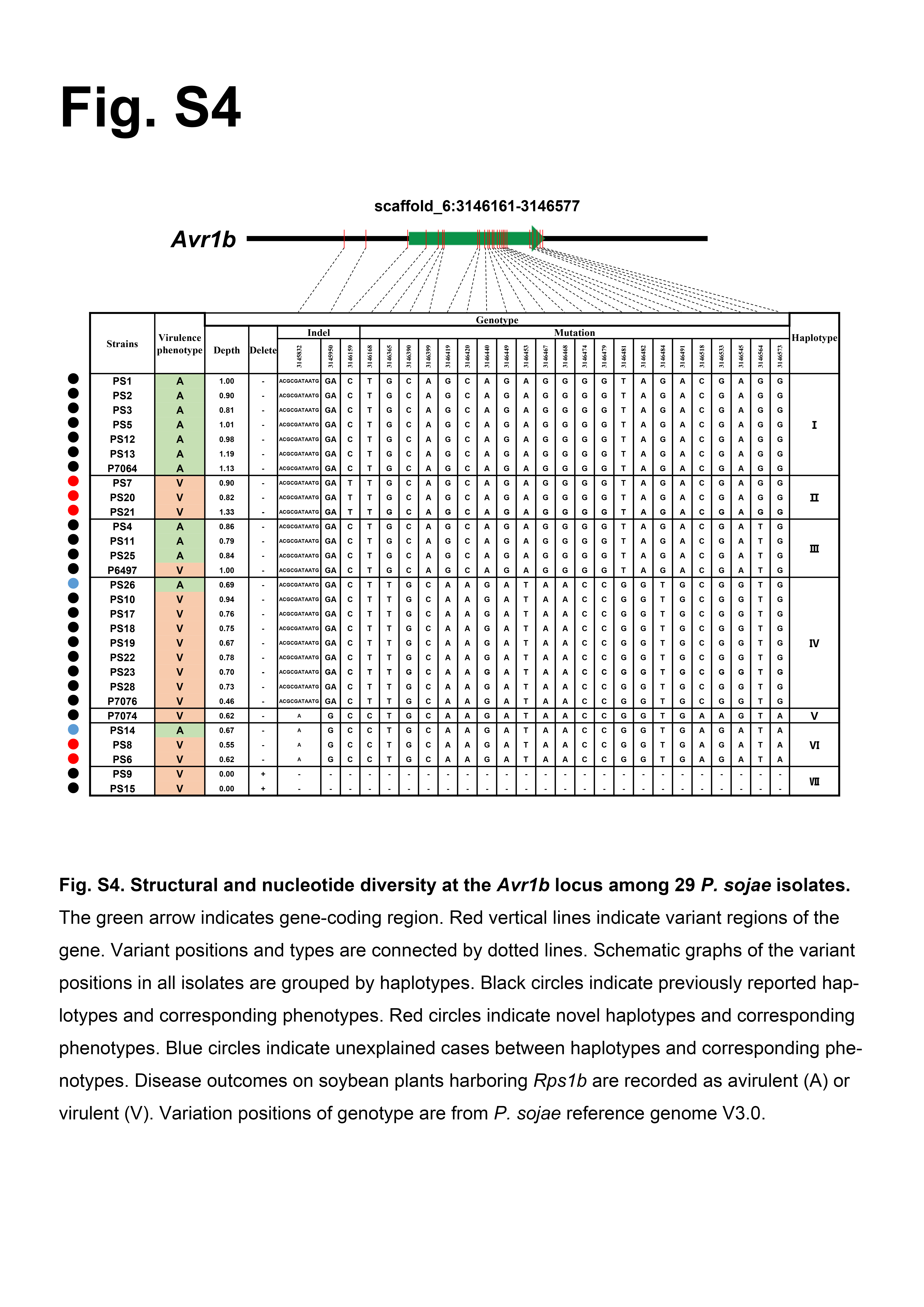

Supplement: Supplementary file 4 [file Image_4.TIF]

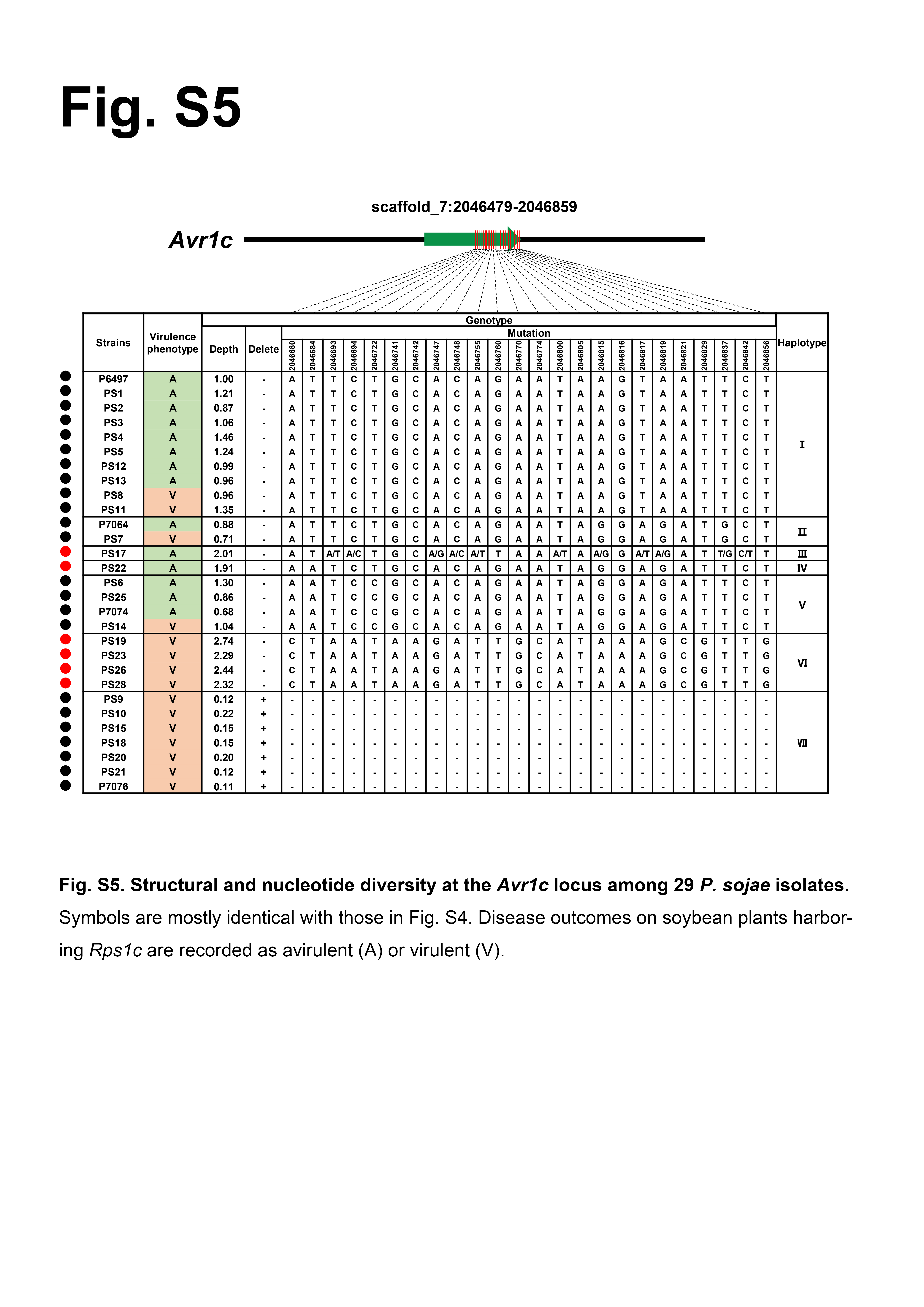

Supplement: Supplementary file 5 [file Image_5.TIF]

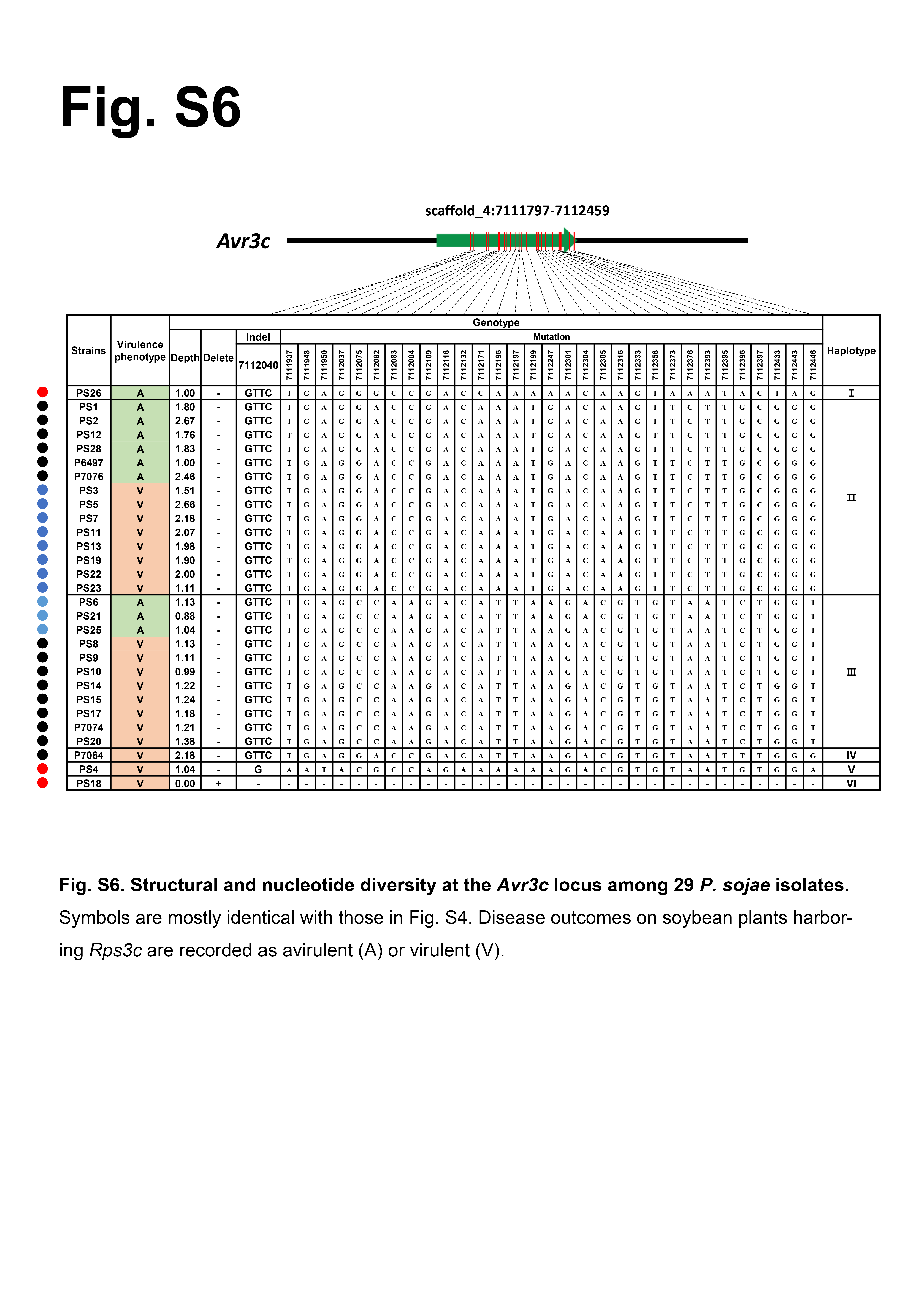

Supplement: Supplementary file 6 [file Image_6.TIF]
